# 5G RF-EMFs Mitigate UV-Induced Genotoxic Stress Through Redox Balance and p38 Pathway Regulation in Skin Cells

**DOI:** 10.3390/antiox15010127

**Published:** 2026-01-19

**Authors:** Ju Hwan Kim, Hee Jin, Kyu Min Jang, Ji Eun Lee, Sanga Na, Sangbong Jeon, Hyung-Do Choi, Jung Ick Moon, Nam Kim, Kyung-Min Lim, Hak Rim Kim, Yun-Sil Lee

**Affiliations:** 1 Department of Pharmacology, College of Medicine, Dankook University, Cheonan-si 31116, Chungnam, Republic of Korea; jhkim731@dankook.ac.kr (J.H.K.); jkm77484@dankook.ac.kr (K.M.J.); 2 College of Pharmacy, Ewha Womans University, Seoul 03760, Republic of Korea; hee_jin@ewha.ac.kr (H.J.); i_mizzi@naver.com (J.E.L.); 001226na@ewhain.net (S.N.); kmlim@ewha.ac.kr (K.-M.L.); 3 Radio and Broadcasting Technology Laboratory, ETRI, Daejeon 34129, Republic of Korea; sbjeon@etri.re.kr (S.J.); choihd@etri.re.kr (H.-D.C.); jungick@etri.re.kr (J.I.M.); 4 School of Electrical and Computer Engineering, Chungbuk National University, Cheongju 28644, Republic of Korea; namkim@chungbuk.ac.kr

**Keywords:** DNA damage, reactive oxygen species, ultraviolet, radiofrequency electromagnetic fields, B16, HaCaT

## Abstract

The biological effects of radiofrequency electromagnetic fields (RF-EMFs) remain an unresolved scientific issue with important societal relevance, particularly in the context of the global deployment of fifth-generation (5G) wireless technologies. The skin is continuously exposed to both RF-EMFs and ultraviolet (UV) radiation, a well-established inducer of oxidative stress and DNA damage, making it a relevant model for assessing combined environmental exposures. In this study, we investigated whether post-exposure to 5G RF-EMFs (3.5 and 28 GHz) modulates ultraviolet A (UVA)-induced genotoxic stress in human keratinocytes (HaCaT) and murine melanoma (B16) cells. Post-UV RF-EMF exposure significantly reduced DNA damage markers, including phosphorylated histone H2AX (γH2AX) foci formation (by approximately 30–50%) and comet tail moments (by 60–80%), and suppressed intracellular reactive oxygen species (ROS) accumulation (by 56–93%). These effects were accompanied by selective attenuation of p38 mitogen-activated protein kinase (MAPK) phosphorylation (reduced by 55–85%). The magnitude of molecular protection was comparable to that observed with N-acetylcysteine treatment or pharmacological inhibition of p38 MAPK. In contrast, RF-EMF exposure did not reverse UV-induced reductions in cell viability or alterations in cell cycle distribution, indicating that its protective effects are confined to early molecular stress-response pathways rather than downstream survival outcomes. Together, these findings demonstrate that 5G RF-EMFs can facilitate recovery from UVA-induced molecular damage via redox-sensitive and p38-dependent mechanisms, providing mechanistic insight into the interaction between modern telecommunication frequencies and UV-induced skin stress.

## 1. Introduction

The maintenance of the structural and functional integrity of DNA is fundamental for genomic stability, cellular viability, and overall organismal health [1]. Ultraviolet (UV) radiation, particularly in the UV-B (280–315 nm) and UV-A (315–400 nm) spectra, is a well-established environmental genotoxic agent [2]. Exposure to UV light induces the formation of cyclobutane pyrimidine dimers (CPDs) and 6–4 photoproducts, which impede DNA replication and transcription [3]. Failure to repair these lesions may result in apoptosis or carcinogenesis [4]. Thus, cells activate several protective mechanisms to mitigate this damage. These include nucleotide excision repair (NER), antioxidant responses, and cell cycle checkpoints [5].

The increasing global deployment of fifth-generation (5G) wireless communication technologies has raised interest in the biological effects of radiofrequency electromagnetic fields (RF-EMFs) [6]. 5G systems operate across a broad spectrum, including the sub-6 GHz band (e.g., 3.5 GHz) and millimeter-wave (mmWave) bands above 24 GHz (e.g., 28 GHz), whose propagation characteristics and tissue penetration depth differ substantially from those of previous generations [7]. Although RF-EMFs are non-ionizing and do not directly break molecular bonds, prolonged or repeated exposure has been reported to influence cellular physiology, particularly through mechanisms involving oxidative stress, calcium signaling, and modulation of gene expression [8]. Importantly, accumulating evidence indicates that the biological effects of RF-EMF exposure are highly context-dependent and vary according to specific exposure parameters, including frequency, specific absorption rate (SAR), and duration, as well as cellular conditions such as the presence of pre-existing stress. In line with this context-dependent framework, recent studies have suggested that RF-EMFs may also exert modulatory or even protective effects on cellular responses to genotoxic stress under certain experimental conditions [9,10]. RF-EMFs modulate DNA repair processes following UV-induced damage, potentially through upregulation of the expression of repair-related genes, attenuation of reactive oxygen species (ROS) production, or stabilization of the cellular redox environment [10,11,12]. These findings challenge the conventional perception of EMFs as purely deleterious stressors, supporting the notion of a nuanced biological interaction contingent on exposure parameters, cellular context, and preexisting DNA damage [13,14].

Despite these emerging insights, the biological interplay between UV-induced DNA damage and subsequent exposure to 5G-relevant EMFs remains unclear. Elucidating whether these interactions are synergistic, antagonistic, or compensatory is particularly important considering the increasing human exposure to complex combinations of physical agents in daily environments [14].

In this study, we aimed to investigate the effect of post-UV exposure to RF-EMFs in the 5G communication bands—specifically at 3.5 GHz and 28 GHz—on DNA damage recovery in mammalian cells. Our findings elucidate the biological effects of RF-EMFs in technologically advanced societies and may contribute to refined health risk assessments under real-world exposure conditions.

## 2. Materials and Methods

### 2.1. Cell Culture

The human keratinocyte cell line HaCaT (CVCL_0038, German Cancer Reserch Center, Heidelberg, Germany) and the murine skin melanoma cell line B16 cells (CVCL_F936, Korea Cell Line Bank, Seoul, Republic of Korea) were cultured in Dulbecco’s modified Eagle’s medium (DMEM; Gibco, Grand Island, NY, USA) supplemented with 10% fetal bovine serum (FBS) and 1% penicillin-streptomycin. Cells were maintained at 37 °C in a humidified incubator with 5% CO_2_.

### 2.2. Antibodies and Reagents

The following antibodies were used: JNK, p-ERK, β-actin (Santa Cruz Biotechnology, Dallas, TX, USA); γH2AX (EMD Millipore, Billerica, MA, USA); p38 MAPK, p-p38 MAPK, p-SAPK/JNK, p44/42 MAPK, α-tubulin (Cell Signaling Technology, Danvers, MA, USA). N-Acetyl-L-cysteine and SB203580 were purchased from Sigma-Aldrich (Sigma-Aldrich, St. Louis, MO, USA).

### 2.3. 3.5 GHz and 28 GHz RF-EMF Exposure Systems

3.5 GHz and 28 GHz 5G exposure system used to conduct controlled in vitro experiments [15,16,17]. RF-EMF exposures were performed at 3.5 and 28 GHz, two frequency bands associated with current and emerging 5G communication services in Korea. While the 3.5 GHz signal was modulated using a 5G NR waveform, the 28 GHz signal was applied as continuous wave (CW) radiation. The compact all-in-one unit integrated all functional components. The exposure chamber was designed to ensure field uniformity, which was validated through planar field mapping. Real-time monitoring of cell temperature was achieved by infrared (IR) camera, and incubator airflow was adjusted during high-power exposures to suppress thermal effects.

### 2.4. Ultraviolet (UV) Irradiation

For UVA irradiation, a Dermalight 80 MED tester (National Biological Corp., Beachwood, OH, USA) equipped with a fluorescent UVA lamp (Philips PL 9W/09; Philips, Eindhoven, The Netherlands) was used. The lamp emitted a spectral range of 320–400 nm, with a peak at approximately ~365 nm. During UVA exposure, the lids of the culture dishes were removed, and the medium was replaced with PBS to avoid the generation of medium-derived photoproducts upon UV illumination. After irradiation (5 J/cm^2^ UVA), PBS was removed and replaced with fresh culture medium. For 3.5 GHz RF exposure, HaCaT cells were exposed to a combination of UVA (365 nm) and UVB (312 nm) at an intensity of 1 J/cm^2^ UVA + 0.03 J/cm^2^ UVB using a UV exposer, Bio-Sun (Vilber Lourmat, Marne-la-Vallée, France). It is designed to accommodate culture Petri dishes or micro-plates inside the UV irradiation system.

### 2.5. Cell Viability

Cell viability against UVA and 5G exposure was determined by 3-(4,5-dimethylthiazol-2-yl)-2,5-diphenyltetrazolium bromide (MTT) assay (Sigma-Aldrich, St. Louis, MO, USA) or a water-soluble tetrazolium salt (WST-1) assay (CellVia, Seoul, Republic of Korea). HaCaT and B16 cells were seeded in 96-well plates at 1 × 10^4^ cells/well. After UV and/or 5G exposure for 24 h, cells were incubated with 100 μL 0.5 mg/mL MTT solution for 4 h or with 10 µL of WST-1 solution in 100 µL of culture medium in each well for 2 h. Absorbance was measured at 540 nm using an ELISA microplate reader (TECAN, Mannedorf, Switzerland) for MTT assay or at 440 nm using a microplate spectrophotometer (Multiskan GO; Thermo Fisher Scientific, Waltham, MA, USA) for WST-1 assay.

### 2.6. Flow Cytometry

Cell death and cell cycle distribution were analyzed by propidium iodide (PI) (Sigma-Aldrich, St. Louis, MO, USA) staining and flow cytometry. Briefly, 2.5 × 10^5^ cells were seeded in 60 mm culture dishes and incubated under standard conditions. After treatment, cells were harvested by trypsinization, centrifuged at 1300 rpm for 3 min, and washed with phosphate-buffered saline (PBS). For cell death analysis, cells were stained with PI (0.5 µg/mL). For cell cycle analysis, cells were fixed in 70% ethanol at −20 °C overnight, washed with PBS, incubated with RNase A (100 µg/mL; Sigma-Aldrich, St. Louis, MO, USA) for 30 min at 37 °C, and stained with PI (50 µg/mL; Sigma-Aldrich, St. Louis, MO, USA). Flow cytometry was performed on a FACSCalibur (BD Biosciences, San Jose, CA, USA), and data were analyzed with CellQuest Pro software (version 5.2, BD Biosciences, San Jose, CA, USA).

### 2.7. Alkaline Comet Assay

DNA strand breaks were assessed by the alkaline comet assay. Briefly, HaCaT and B16 cell suspensions (1 × 10^4^ cells/mL) were mixed with low-melting-point agarose (LMAgarose; Trevigen, Gaithersburg, MD, USA) and evenly spread onto microscope slides. The slides were immersed in chilled lysis solution (Trevigen, Gaithersburg, MD, USA) at 4 °C for 1 h in the dark, incubated in alkaline electrophoresis buffer (pH > 13) for 20 min, and electrophoresed at 25 V for 30 min. Slides were neutralized with Tris-HCl buffer (pH 7.5) and stained with SYBR Gold (Trevigen, Gaithersburg, MD, USA). Comets were visualized using a Zeiss fluorescence microscope (Carl Zeiss, Oberkochen, Germany) at 5× magnification. At least 50 comets were analyzed for each sample, and DNA damage was quantified as olive tail moment using Comet Assay Software (version Komet 5.5, Andor Technologies, Abingdon, UK). Each assay was performed in triplicate across a minimum of three independent experiments.

### 2.8. ROS Measurement

The intracellular ROS levels were measured using the DCFDA/H2DCFDA Cellular ROS assay kit (Abcam, Cambridge, UK). HaCaT and B16 cells were seeded in 24-well plates at 4 × 10^4^ cells/well. After treatment, cells were washed with PBS and incubated with 20 µM 2’,7’-dichlorofluorecin diacetate (DCFDA) for 30 min at 37 °C in the dark. Images were acquired with a Zeiss Apotome (Carl Zeiss, Oberkochen, Germany).

### 2.9. Immunoblotting

Cells were lysed with RIPA buffer (Biosesang, Incheon, Republic of Korea), and protein concentrations were determined by the Bradford assay (Bio-Rad, Hercules, CA, USA). Equal amounts of protein were separated by SDS-PAGE (6–15%) and transferred to nitrocellulose membranes. After blocking with 5% skim milk in PBS-T, membranes were incubated overnight at 4 °C with primary antibodies, followed by horseradish peroxidase-conjugated secondary antibodies (Santa Cruz Biotechnology, Dallas, TX, USA). Immunoreactive bands were detected using enhanced chemiluminescence (EzWestLumi, Taito-ku, Tokyo, Japan) and visualized with a ChemiDoc imaging system (Bio-Rad, Hercules, CA, USA). Protein band intensities were quantified with ImageJ software (version 1.54p, NIH, Bethesda, MD, USA).

### 2.10. Immunofluoresce (IF)

Cells were cultured on chamber slides (SPL Life Sciences, Pocheon-si, Republic of Korea) and fixed in 4% paraformaldehyde for 30 min at room temperature. After washing with phosphate-buffered saline (PBS), fixed cells were permeabilized with 0.1% Triton X-100 and incubated for 30 min with 1% bovine serum albumin to block nonspecific antibodies. For immunofluorescence staining, cells were incubated with γH2AX (1:1000) for 3 h at 4 °C overnight. Next, samples were incubated with Alexa-488 conjugated anti-mouse (1:500) secondary antibodies for 1 h at room temperature in the dark. Nucleus was counterstained with DAPI (Sigma-Aldrich, St. Louis, MO, USA) and all immunofluorescence images were captured by a Zeiss Apotome (Carl Zeiss, Oberkochen, Germany).

### 2.11. Statistical Analysis

Data are presented as the mean ± standard deviation (SD) and were analyzed using GraphPad Prism 9 (GraphPad Software Inc., San Diego, CA, USA). Statistical significance was determined by one-way ANOVA followed by Dunnett’s multiple comparisons test, Tukey’s post hoc test, or Student’s *t*-test as appropriate. *p*-values < 0.05 were considered statistically significant.

## 3. Results

### 3.1. Effects of 28 GHz RF-EMF and UVA Exposure in HaCaT and B16 Cells

**Figure 1:**
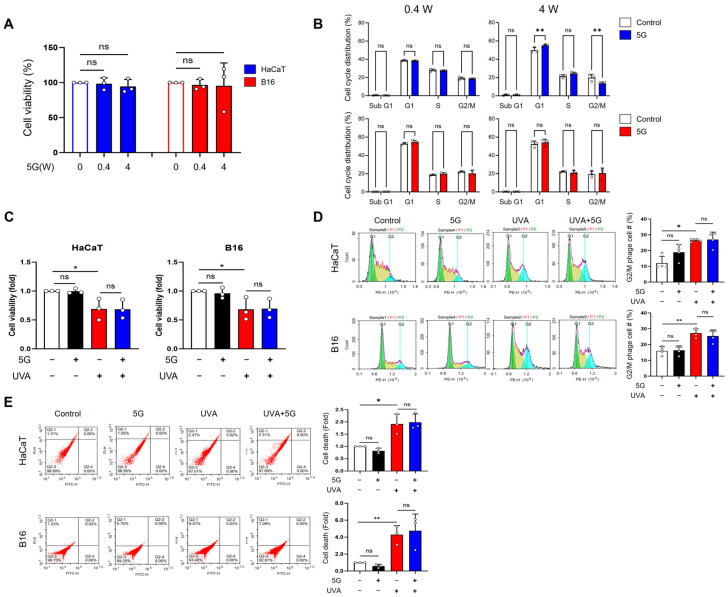
Effects of 28GHzRF-EMF and UVA exposure on HaCaT and B16 cells (**A**) HaCaT and B16 cells were exposed to 0, 0.4, or 4 W/kg of 28 GHz RF-EMF for 24 h, and cell viability was assessed using the MTT assay (*n* = 3). (**B**) The distribution of cell cycle phases in HaCaT and B16 cells after 0.4 or 4 W/kg RF-EMF exposure was examined by PI staining followed by flow cytometry (*n* = 3). (**C**) Following UVA (5 J/cm^2^) and 28 GHz RF-EMF (4.0 W/kg, 24 h) exposure, HaCaT and B16 cell numbers were counted to determine cell viability (*n* = 3). (**D**) Changes in the cell cycle distribution of HaCaT and B16 cells were evaluated after combined UVA and RF-EMF treatment using PI staining and flow cytometry (*n* = 4). Different colors represent cell cycle phases: G0/G 1(green), S (yellow), and G2/M (blue). The graph shows changes (%) in the G2/M phase only during the cell cycle. (**E**) The extent of cell death in HaCaT and B16 cells after UVA and RF-EMF exposure was monitored via PI staining and flow cytometry (*n* = 3). The graph shows changes (fold) in the cell death of HaCaT and B16 cells. The *n* value indicates the number of independent biological replicates performed on different days using separate cell passages. The data indicate the mean  ±  SD. Levels of statistical significance were evaluated using one-way ANOVA or unpaired Student’s *t*-tests; * *p*  <  0.05, ** *p*  <  0.01 vs. control; ns, not significant. Group designations are indicated by bar colors: white (control), black (RF-EMF only), red (UV only), and blue (combined UV and RF-EMF).

The effects of RF-EMF exposure and UV irradiation on HaCaT human keratinocytes and B16 murine melanoma cells were assessed based on cell viability, cell cycle distribution, and cell death. For 28 GHz RF-EMF, exposure at 0.4 or 4 W/kg did not significantly affect the viability of either HaCaT or B16 cells (Figure 1A). Cell cycle analysis revealed that 0.4 W/kg RF-EMF caused no detectable changes in either cell type, whereas 4 W/kg exposure led to an increase in the G1 and S phases and a decrease in the G2/M phase in HaCaT cells; B16 cells remained unaltered (Figure 1B).

In HaCaT and B16 cells exposed to 28 GHz RF-EMF (4 W/kg) and UVA irradiation (HaCaT: 5 J/cm^2^; B16: 5 J/cm^2^), UVA alone significantly reduced cell viability by approximately 35% in both cell lines, whereas RF-EMF alone had no effect. Combined RF-EMF and UVA exposure did not further decrease cell survival compared to UVA exposure alone (Figure 1C). Similarly, UVA exposure induced a nearly two-fold increase in the G2/M population in both cell types, whereas RF-EMF alone did not alter cell cycle progression (Figure 1D). Cell death analysis confirmed that UVA, but not RF-EMF, markedly increased cell death in HaCaT (2-fold) and B16 (nearly 4-fold) cells (Figure 1E).

### 3.2. Effects of 3.5 GHz RF-EMF and UV Exposure in HaCaT and B16 Cells

**Figure 2:**
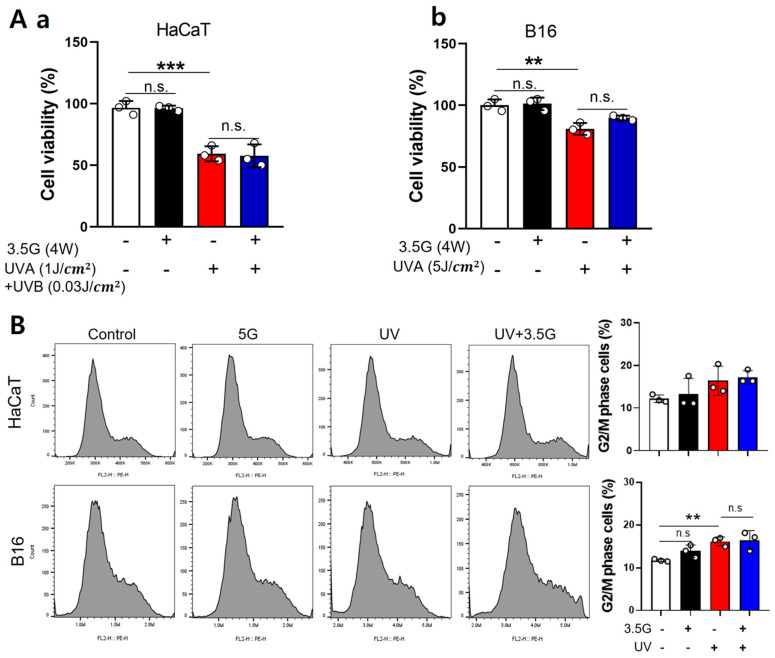
Effects of UV and 3.5 GHz RF-EMF exposure on HaCaT and B16 cells. (**A**) Cell viability in HaCaT and B16 cells after combined UV and 3.5 GHz RF-EMF exposure. The total number of HaCaT (**a**) and B16 (**b**) cells was counted following exposure to UV (HaCaT: 1 J/cm^2^ UVA + 0.03 J/cm^2^ UVB; B16: 5 J/cm^2^ UVA), with or without subsequent 3.5 GHz RF-EMF treatment (4 W/kg for 24 h) (*n* = 3). (**B**) G2/M phase distribution in HaCaT and B16 cells after combined UV and 3.5 GHz RF-EMF exposure, analyzed using the Cell Muze system to evaluate changes in cell cycle progression (*n* = 3). The value *n* = 3 indicates three independent biological replicates performed on different days using separate cell passages. The data indicate the mean  ±  SD. Levels of statistical significance were evaluated using unpaired Student’s *t*-tests; ** *p*  <  0.01, *** *p*  <  0.001 vs. control; ns, not significant. Group designations are indicated by bar colors: white (control), black (RF-EMF only), red (UV only), and blue (combined UV and RF-EMF).

For 3.5 GHz RF-EMF, HaCaT and B16 cells were exposed to 4 W/kg RF-EMF, followed by UV irradiation (HaCaT: 1 J/cm^2^ UVA + 0.03 J/cm^2^ UVB; B16:5 J/cm^2^ UVA). RF-EMF exposure alone did not affect cell viability, whereas UV exposure alone significantly reduced the survival of both cell types by approximately 40% and 20%, respectively. Combined RF-EMF and UV exposure did not further decrease cell viability compared to UV exposure alone (Figure 2A). Cell cycle analysis using the Cell Muze system revealed a significant increase in the G2/M (gap 2/mitosis) phase following UV exposure (a 37% increase) in B16 cells, whereas HaCaT cells exhibited a similar increasing trend without statistical significance; RF-EMF alone did not induce any notable changes (Figure 2B). Overall, the common findings for 28 GHz and 3.5 GHz RF-EMF exposures were that RF-EMF alone has a minimal effect on cell viability or cell cycle distribution. In contrast, UV irradiation consistently reduced cell survival and altered cell cycle progression. These results indicate that RF-EMF does not exacerbate UV-induced cellular effects.

### 3.3. RF-EMF Exposure at 28 and 3.5 GHz Mitigates UVA-Induced DNA Damage Responses

**Figure 3:**
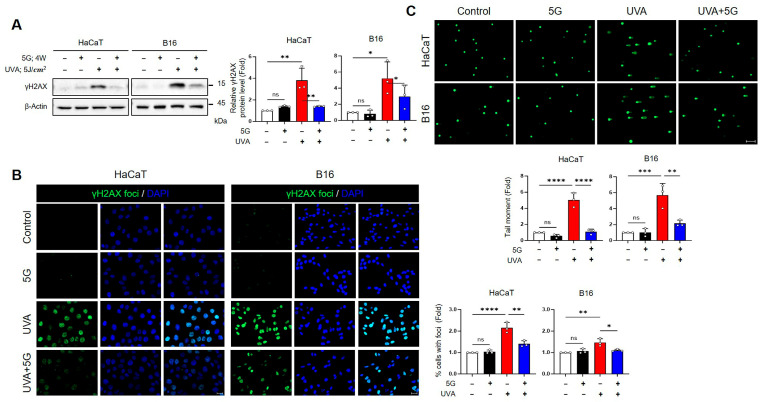
Effects of 28 GHz RF-EMF on UVA-induced DNA damage in HaCaT and B16 cells. (**A**) Western blot analysis of γH2AX (Ser139) in HaCaT and B16 cells after 28 GHz RF-EMF (4 W/kg, 24 h) and/or UVA (5 J/cm^2^) exposure, illustrating changes in DNA damage marker expression. The graph shows changes (fold) in the relative γH2AX protein level of HaCaT and B16 cells (*n* = 3). (**B**) Representative images of γH2AX foci in HaCaT and B16 cells detected by immunocytochemistry after 28 GHz RF-EMF and/or UVA exposure (*n* = 3). The graph shows % cells with γH2AX foci (fold) in HaCaT and B16 cells. (**C**) DNA damage assessed by alkaline comet assay, showing tail formation in HaCaT and B16 cells following 28 GHz RF-EMF and/or UVA exposure. The graph shows the tail moment (fold) in HaCaT and B16 cells. The value *n* = 3 indicates three independent biological replicates performed on different days using separate cell passages. The data indicate the mean  ±  SD. Levels of statistical significance were evaluated using one-way ANOVA or unpaired Student’s *t*-tests; * *p*  <  0.05, ** *p*  <  0.01, *** *p*  <  0.001, **** *p*  <  0.0001 vs. control; ns, not significant. Group designations are indicated by bar colors: white (control), black (RF-EMF only), red (UV only), and blue (combined UV and RF-EMF).

**Figure 4:**
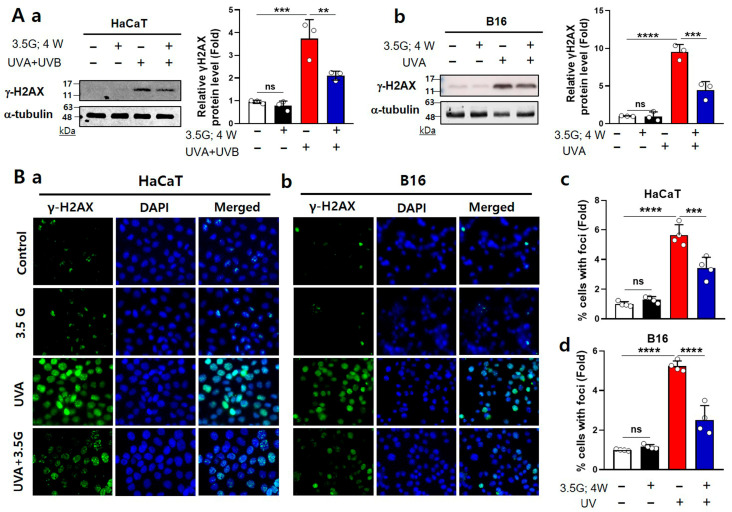
Effects of 3.5 GHz RF-EMF on UVA-induced DNA damage in HaCaT and B16 cells. (**A**) Immunoblotting of γH2AX (Ser139) in HaCaT (**a**) and B16 (**b**) cells following UV (HaCaT: 1 J/cm^2^ UVA + 0.03 J/cm^2^ UVB; B16: 5 J/cm^2^ UVA) and 3.5 GHz RF-EMF (4 W/kg, 24 h) exposure (*n* = 3). Band intensities were normalized to α-tubulin. (**B**) Fluorescence images of γH2AX foci in HaCaT (**a**) and B16 (**b**) cells after UV and 3.5 GHz RF-EMF treatments (*n* = 4). Foci intensity was quantified using ImageJ (HaCaT: (**c**); B16: (**d**)). The *n* value indicates the number of independent biological replicates performed on different days using separate cell passages. The data indicate the mean  ±  SD. Levels of statistical significance were evaluated using unpaired Student’s *t*-tests; ** *p*  <  0.01, *** *p*  <  0.001, **** *p*  <  0.0001 vs. control; ns, not significant. Group designations are indicated by bar colors: white (control), black (RF-EMF only), red (UV only), and blue (combined UV and RF-EMF).

To evaluate the effect of RF-EMF on UVA-induced DNA damage, we assessed both 28 GHz and 3.5 GHz exposures in HaCaT keratinocytes and B16 melanoma cells. RF-EMF exposure alone (4 W/kg, 24 h) did not affect DNA damage markers, whereas UV exposure (HaCaT: 28 GHz 1 J/cm^2^ UVA + 0.03 J/cm^2^ UVB; B16: 5 J/cm^2^ UVA) induced considerable DNA damage responses. Western blotting revealed that UVA strongly increased γH2AX protein expression in both cell types, whereas subsequent RF-EMF exposure significantly reduced γH2AX expression toward baseline levels by approximately 63% (HaCaT) and 43% (B16) at 28 GHz, and by 44% (HaCaT) and 53% (B16) at 3.5 GHz (28 GHz: Figure 3A; 3.5 GHz: Figure 4A). Immunofluorescence staining showed that UVA exposure led to the formation of abundant γH2AX foci, which was markedly suppressed by RF-EMF by an average of 37% (HaCaT) and 30% (B16) at 28 GHz, and 40% (HaCaT) and 52% (B16) at 3.5 GHz, respectively (28 GHz: Figure 3B; 3.5 GHz: Figure 4B). Consistently, the alkaline comet assay demonstrated that UVA-induced DNA strand breaks, reflected by increased tail moments, were substantially diminished by approximately 78% (HaCaT) and 62% (B16) following RF-EMF treatment (28 GHz: Figure 3C). Collectively, these findings demonstrate that both 28 GHz and 3.5 GHz RF-EMF exposures effectively attenuate UVA-induced DNA damage in keratinocytes and melanoma cells.

### 3.4. RF-EMFs Attenuate UV-Induced ROS Production and p38 MAPK Activation in HaCaT and B16 Cells at 28- and 3.5-GHz Exposure

**Figure 5:**
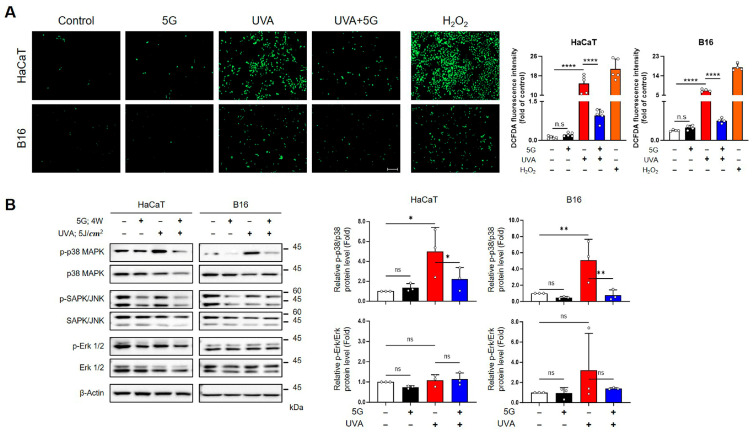
Effects of 28 GHz RF-EMF on UVA-induced ROS generation and MAPK activation in HaCaT and B16 cells. (**A**) Intracellular ROS levels were measured using the fluorescent probe 2’,7’-dichlorofluorescin diacetate (DCFDA) fluorescence. HaCaT and B16 cells were exposed to UVA (5 J/cm^2^) with or without subsequent 28 GHz RF-EMF exposure (4 W/kg, 24 h) (HaCaT *n* = 5 and B16 *n* = 4). Hydrogen peroxide (H_2_O_2_) treatment was used as a positive control. DCFDA fluorescence intensities were analyzed using ImageJ. (**B**) Western blot analysis of phosphorylated MAPK proteins (p38, JNK, ERK1/2) in HaCaT and B16 cells following UVA and/or 28 GHz RF-EMF exposure (*n* = 3). The *n* value indicates the number of independent biological replicates performed on different days using separate cell passages. The bars indicate the mean  ±  SD. Statistically significant levels were evaluated using one-way ANOVA or unpaired Student’s *t*-tests * *p*  <  0.05, ** *p*  <  0.01, **** *p*  <  0.0001 vs. control; ns, not significant. Group designations are indicated by bar colors: white (control), black (RF-EMF only), red (UV only), and blue (combined UV and RF-EMF).

**Figure 6:**
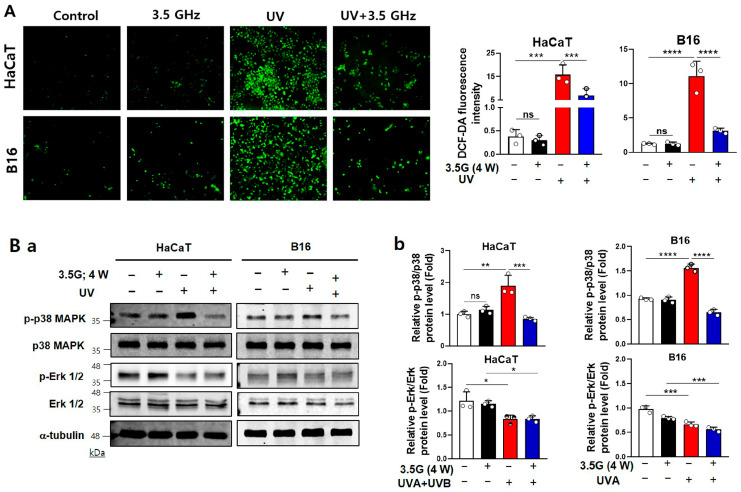
Effects of UVA and 3.5 GHz RF-EMF on ROS production and MAPK signaling in HaCaT and B16 cells. (**A**) Intracellular ROS levels were assessed using DCFDA fluorescence in HaCaT and B16 cells exposed to UVA (HaCaT: 1 J/cm^2^ UVA + 0.03 J/cm^2^ UVB; B16: 5 J/cm^2^ UVA), followed by 3.5 GHz RF-EMF (4.0 W/kg, 24 h) (*n* = 3). DCFDA fluorescence was quantified using ImageJ. (**B**) Western blot analysis of phosphorylated MAPK proteins. (**a**) Representative immunoblots of γH2AX, phospho-p38 (Thr180/Tyr182), total p38, phospho-ERK1/2, and total ERK1/2 in HaCaT cells (*n* = 3). (**b**) Band density analysis of phospho-p38 and phospho-ERK1/2 normalized to their respective total protein levels. The value *n* = 3 indicates three independent biological replicates performed on different days using separate cell passages. The data indicate the mean  ±  SD. Levels of statistical significance were evaluated using unpaired Student’s *t*-tests; * *p*  <  0.05, ** *p*  <  0.01, *** *p*  <  0.001, **** *p*  <  0.0001 vs. control; ns, not significant. Group designations are indicated by bar colors: white (control), black (RF-EMF only), red (UV only), and blue (combined UV and RF-EMF).

Intracellular ROS levels were measured by DCFDA fluorescence to determine whether RF-EMFs modulate UVA-induced oxidative stress. UVA exposure led to a robust increase in ROS intensity in both HaCaT and B16 cells. Specifically, UVA irradiation (5 J/cm^2^) caused marked ROS elevation under the 28 GHz condition, whereas HaCaT (1 J/cm^2^ UVA + 0.03 J/cm^2^ UVB) and B16 (5 J/cm^2^ UVA) cells displayed similar ROS increases under the 3.5 GHz condition. Importantly, subsequent RF-EMF exposure (4 W/kg, 24 h) significantly suppressed UVA-induced ROS accumulation, restoring levels to near baseline in B16 cells and markedly reducing levels in HaCaT cells. In contrast, RF-EMF exposure alone did not alter ROS levels (Figure 5A and Figure 6A).

We subsequently investigated whether RF-EMFs affect UVA-induced MAPK pathway activation. Under both frequencies, UVA irradiation strongly induced p38 MAPK phosphorylation in HaCaT and B16 cells. RF-EMF exposure consistently attenuated this p38 activation, bringing phospho-p38 levels close to those of untreated controls by an average of 55% (HaCaT) and 85% (B16) at 28 GHz, and 55% (HaCaT) and 58% (B16) at 3.5 GHz, respectively (Figure 5B and Figure 6B). In contrast, UVA exposure only significantly decreased phospho-ERK1/2 under the 3.5 GHz condition in both HaCaT and B16 cells, and RF-EMF did not reverse this reduction. No significant changes were detected in total p38 or ERK1/2 expression across all conditions. Similarly, phospho-JNK levels remained unchanged (Figure 5B and Figure 6B). These findings demonstrate that both 28 and 3.5 GHz RF-EMFs effectively suppress UVA-induced ROS overproduction and selectively downregulate p38 MAPK activation in HaCaT and B16 cells.

### 3.5. Comparative Effects of RF-EMF, Antioxidant, and p38 Inhibition on UVA-Induced DNA Damage and Signaling in HaCaT and B16 Cells

UVA irradiation markedly increased DNA damage and p38 activation in HaCaT and B16 cells, as reflected by γH2AX accumulation and elevated phospho-p38 levels. Subsequent exposure to RF-EMF (28 or 3.5 GHz) consistently suppressed these UVA-induced responses. The extent of reduction was comparable to that observed with the antioxidant N-acetylcysteine (NAC) (28 GHz, Figure S1A,B) or with pharmacological inhibition of p38 (28 GHz and 3.5 GHz, Figures S1C,D and S2A,B). These findings indicate that RF-EMF exposure exerts protective effects against UVA-induced DNA damage through a mechanism that functionally converges with selective p38 inhibition.

## 4. Discussion

Recent studies have debated the potential toxicity or harmlessness of RF-EMFs in cells. Jin et al. (2021) [10] reported significantly reduced DNA DSBs in HaCaT and B16 cells exposed to 1.762 GHz LTE-EMF (8 W/kg), along with enhanced DNA repair after exposure to ionizing radiation and bleomycin. Based on these findings, the present study investigated the protective effects of 5G RF-EMFs (3.5 GHz and 28 GHz) on UV-induced DNA damage in skin cells. In this study, we explored the mechanism by which 5G RF-EMFs facilitate DNA repair and recovery in human keratinocytes (HaCaT) and murine melanoma cells (B16). Our findings provide insights into the potential effects of these RF-EMFs on skin cells.

In the present study, post-UV exposure to 5G-relevant RF-EMFs (3.5 GHz and 28 GHz) attenuated UVA-induced genotoxic stress in HaCaT and B16 cells. RF-EMF treatment reduced the expression of canonical DNA-damage markers (γH2AX) and/or comet-assay tail moments (Figure 3A–C and Figure 4AB) and mitigated ROS overproduction (Figure 5A and Figure 6A). It also selectively suppressed p38 MAPK phosphorylation (Figure 5B and Figure 6B). In contrast, no RF-EMF-related changes were observed in the UV-driven increases in cell death or G2/M cell cycle arrest (Figure 1D,E and Figure 2B), indicating that these endpoints were dominated by UV exposure. The protective effect of RF-EMFs was evident mainly at the level of oxidative stress and DNA damage signaling (Figure 3, Figure 4, Figure 5 and Figure 6), and its magnitude was comparable to that achieved with the antioxidant NAC and pharmacological p38 MAPK inhibition (Figures S1A–D and S2A,B). Taken together, these results show that RF-EMF exposure functionally converges with ROS scavenging and p38 suppression to facilitate the recovery from UVA-induced DNA damage. A potential limitation of this study is the relatively small sample size (*n* = 3–5), which may limit statistical power, particularly for the detection of modest effect sizes. To enhance the reliability of the data, each experiment was performed using independent biological replicates, including different cell passages cultured on separate days and independent UV and RF-EMF exposure sessions. Consistent with effect sizes reported in previous studies, key protective markers such as reduced ROS levels, decreased p38 MAPK phosphorylation, and attenuated γ-H2AX foci formation reached statistical significance across these independent replicates, suggesting that the RF-EMF–associated effects on early stress-response signaling pathways are reproducible and biologically relevant. In contrast, downstream endpoints such as cell viability did not show statistically significant changes. The consistency of these null findings across independent biological replicates suggests that large RF-EMF–mediated effects on these parameters are unlikely under the present experimental conditions. However, smaller effects cannot be excluded due to limited statistical power and should be interpreted with caution. In this context, the dominant influence of UV exposure may overshadow any subtle modulatory effects of RF-EMF on these downstream cellular outcomes.

Although RF-EMF treatment effectively facilitated molecular recovery from UV-induced DNA lesions (Figure 1C and Figure 2A), it did not rescue the reduction in cell viability. This limitation is explained by the irreversible nature of programmed cell death signaling once apoptosis has been initiated; if UV irradiation triggers apoptotic cascades within minutes, later restoration of DNA integrity will not reverse executioner-caspase activation or the cell death trajectory [18,19,20]. In addition, persistent G2/M arrest, even when DNA damage is partially repaired, can lead to functional decline and failure to re-enter proliferation, thereby sustaining low viability [21,22,23]. Therefore, although RF-EMFs support lesion recovery at the molecular level, they cannot fully counteract early apoptosis or prolonged checkpoint arrest triggered by UV irradiation [13,24,25].

Haidar et al. [26] tested exposure to 5G-modulated 3.5 GHz fields up to 4 W/kg in vitro and reported no induction of oxidative stress or impairment of DNA repair efficiency in human skin cells. The apparent discrepancy can be explained by differences in UV wavelengths and lesion types (UVB/CPD vs. UVA/oxidative lesions), timing of RF exposure relative to UV-induced damage, and the specific endpoints measured. Haidar et al. [26] employed a relatively mild UVB dose (20 mJ/cm^2^) optimized to preserve high cell viability and maintain an efficient intrinsic repair capacity in HaCaT cells. These conditions may have obscured any additional or synergistic effects of the RF-EMFs. In contrast, our study applied a combined UV regimen consisting of UVB (30 mJ/cm^2^) and UVA (1 J/cm^2^), which elicited more pronounced cellular damage and lower baseline survival than the milder 20 mJ/cm^2^ UVB condition. Importantly, similar recovery-promoting and antioxidant effects were also observed under 28 GHz exposure following UVA-only (5 J/cm^2^) irradiation, suggesting that the beneficial effect of RF-EMFs is not confined to a single frequency band but extends across 5G-relevant spectra. We propose that this higher initial lesion burden, particularly UVA-induced oxidative stress (Figure 3, Figure 4, Figure 5 and Figure 6), establishes a cellular environment in which endogenous repair mechanisms are challenged, thereby revealing the therapeutic or assistive potential of 3.5 and 28 GHz RF-EMFs. Haidar et al. [26] reported that a 3.5 GHz RF-EMF does not intrinsically provoke oxidative stress or impair DNA repair under moderate conditions or in the absence of stress. Similarly, our data demonstrate that RF-EMF exposure can facilitate molecular recovery through redox- and p38-sensitive signaling pathways under conditions of pre-existing UVA-induced oxidative lesions that overwhelm repair capacity.

Mechanistically, the two nonmutually exclusive routes plausibly account for our results. First, RF-EMF exposure may upregulate or mobilize antioxidant defenses (e.g., superoxide dismutase (SOD), catalase (CAT), and glutathione-dependent systems), thereby lowering net ROS production after UVA and reducing the oxidative conversion of base lesions to more complex DSB-forming lesions [12,27,28]. Our DCF-DA data and the equivalence of RF-EMF and NAC in reducing γH2AX/tail moments support this postulation [12,13,29,30]. Second, RF-EMF might modulate signaling cascades that regulate DNA damage responses and repair (notably p38), thereby accelerating lesion recognition/processing or improving the balance of repair pathway engagement (NER/base excision repair (BER) vs. DSB repair) to reduce the lesion burden before DSB conversion occurs [31,32,33,34,35]. Jin et al. [10] previously reported LTE-EMF-associated reductions in γH2AX and changes in repair-gene expression in skin models; the present findings extend this protective phenotype to UVA and 5G bands.

The specificity of the RF-EMF effect is reproducible. Phospho-p38 was consistently downregulated, phospho-JNK remained unchanged, and phospho-ERK1/2 showed a frequency-dependent behaviour [36,,37,38]. This pattern is against non-specific global pathway shutdown and suggests targeted, redox-sensitive modulation of stress kinases, consistent with the functional equivalence of p38 inhibition [29,39,40,41].

Our findings are consistent with those of previous studies in which protective or adaptive responses to RF exposure have been reported in some experimental paradigms [9,10,26,34,42] and neutral or pro-oxidant outcomes have been reported in others [11,12,13,43,44,45,46]. These differences highlight the dependence of results on frequency, modulation, SAR, timing relative to other stressors, and cellular context [43,47,48,49]. In the present study, we employed parallel post-UV exposure paradigms at 28 GHz and 3.5 GHz with matched SAR (4 W/kg) and well-defined UVA/UVB dosing. Our experimental conditions demonstrate that RF-EMF exposure can mitigate UVA-induced oxidative signaling through an ROS–p38-dependent pathway in skin cells.

The hypotheses that RF-EMFs enhance antioxidant defenses and accelerate DNA repair are indirect; they are inferred solely from the observed reduction in ROS and γH2AX levels (Figure 3, Figure 4, Figure 5 and Figure 6). Therefore, future studies should incorporate rigorous biochemical and molecular assays to directly verify the proposed mechanisms. Specifically, direct quantification of the functional activity of key antioxidant enzymes, including SOD, CAT, and glutathione peroxidase, is essential, along with precise measurement of the cellular redox state based on the GSH (reduced glutathione) to GSSG (oxidized glutathione) ratio [26,50,51,52]. Furthermore, the quantification of DNA repair proteins and checkpoint kinases, such as XPA/XPC, OGG1, APE1, RAD51, MRE11, and ATM/ATR, is crucial for defining the specific repair pathways modulated by RF-EMF [5,26,48,53,54,55,56,57].

## 5. Conclusions

In conclusion, post-UV exposure to RF-EMFs at 3.5 and 28 GHz (5G frequency bands) reduces UVA-induced ROS overproduction, selectively attenuates p38 activation, and decreases γH2AX accumulation and DNA fragmentation. This protective phenotype mirrors that observed following NAC treatment and p38 inhibition, suggesting that RF-EMFs act via redox-sensitive, p38-dependent mechanisms and/or facilitate DNA repair processes. Importantly, our results demonstrate that RF-EMFs at 3.5 and 28 GHz confer molecular protection in in vitro skin models, highlighting the potential for further mechanistic studies.

## Data Availability

The original contributions presented in this study are included in the article/Supplementary Material. Further inquiries can be directed to the corresponding author.

## Supplementary Materials

The following supporting information can be downloaded at: https://www.mdpi.com/article/10.3390/antiox15010127/s1, Figure S1: Comparative effects of N-Acetyl Cysteine (NAC) and p38 inhibitor on 28 GHz RF-EMF-induced responses after UVA damage in HaCaT cells and B16 cells.; Figure S2: Comparing the impact of a p38 inhibitor on HaCaT cell reactions to 3.5 GHz RF-EMF following UV exposure.

## Abbreviations

APE1	Apurinic/apyrimidinic endonuclease 1
ATM	Ataxia-telangiectasia mutated
ATR	Ataxia-telangiectasia and RAD3-related
BER	Linear dichroism
CAT	Catalase
CPD	Cyclobutane pyrimidine dimer
DSB	Double-strand break
5G	Fifth-Generation
GSH	reduced Glutathione
GSSG	oxidized Glutathione
LTE	Long-term evolution
MRE11	Meiotic recombination 11
NER	Nucleotide excision repair
NAC	N-acetylcysteine
OGG1	8-oxoguanine DNA glycosylase
RAD51	Recombinant DNA repair protein 51
RF-EMF	Radiofrequency-electromagnetic fields
SAR	Specific absorption rate
SOD	Superoxide dismutase
UV	Ultraviolet
XPA	Xeroderma pigmentosum group A
XPC	Xeroderma pigmentosum group C
