# Supplementary material for: 5G RF-EMFs Mitigate UV-Induced Genotoxic Stress Through Redox Balance and p38 Pathway Regulation in Skin Cells
Source: Antioxidants (Basel). 2026 Jan 19;15(1):127. doi: 10.3390/antiox15010127 (PMC12837458; doi:10.3390/antiox15010127)
Supplement: Supplementary file 1 [file antioxidants-15-00127-s001.zip › antioxidants-4040115-supplementary.pdf]

# 5G RF-EMFs Mitigate UV-Induced Genotoxic Stress Through Redox Balance and p38 Pathway Regulation in Skin Cells

**Ju Hwan Kim [1,#], Hee Jin [2,#], Kyu Min Jang [1], Ji Eun Lee[2], Sanga Na[2], Sangbong Jeon [3], Hyung-Do Choi [3], Jung Ick Moon[3], Nam Kim [3], Kyung-Min Lim [2], Hak Rim Kim [1] * and Yun-Sil Lee [2] ***

## Supplementary Materials

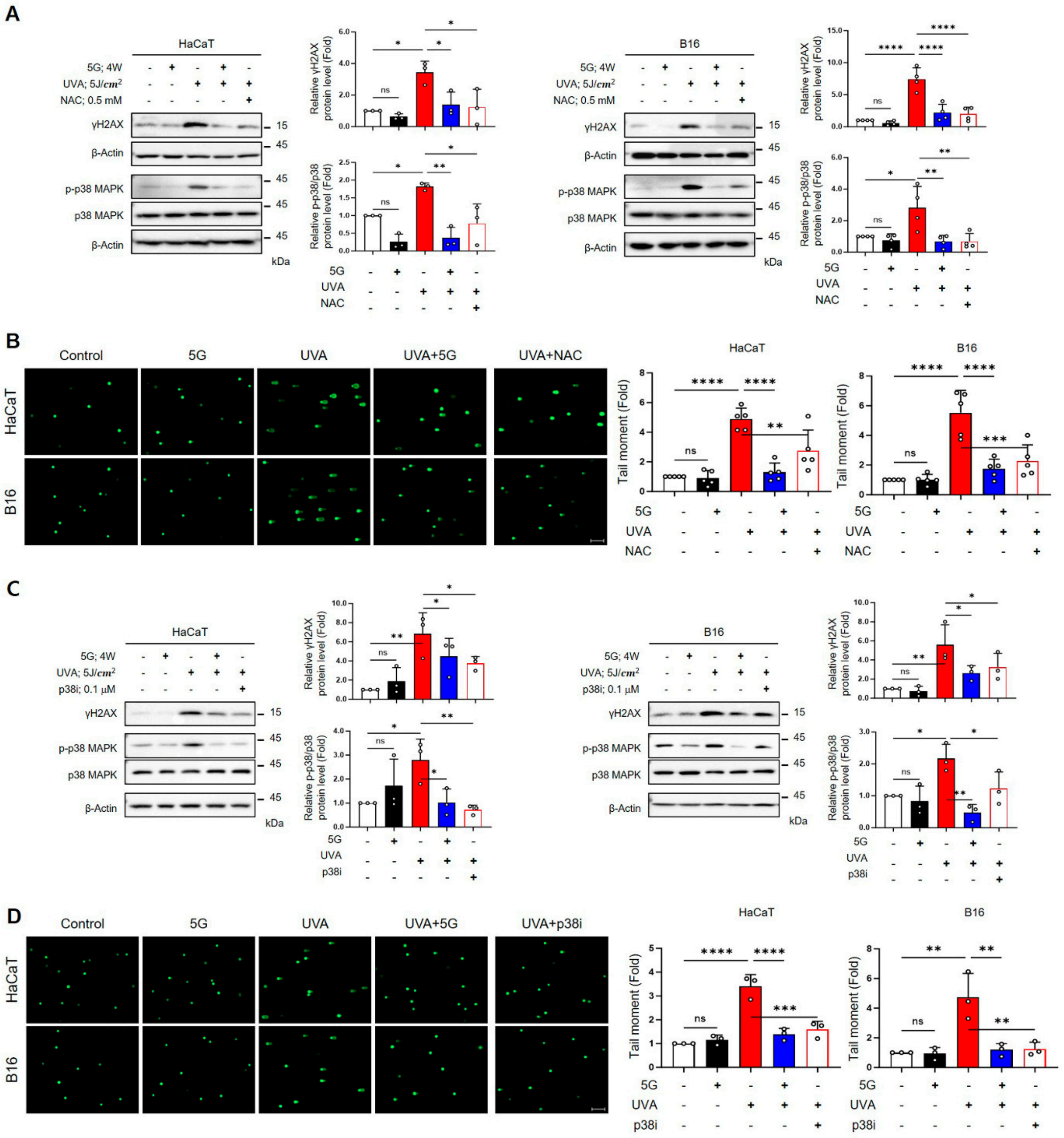

**Figure S1. Comparative effects of N-Acetyl Cysteine (NAC) and p38 inhibitor on 28 GHz RF-EMF-induced responses after UVA damage in HaCaT cells and B16 cells. A**. Western blot analysis of γH2AX and phospho-p38 in HaCaT (**a**) and B16 (**b**) cells following UVA, RF-EMF, and NAC treatment ($n$=3). **B**. DNA damage assessment by alkaline comet assay in HaCaT and B16 cells under the same conditions with NAC treatment ($n$=5). **C**. Western blot analysis of γH2AX and phosphorylated p38 in HaCaT (**a**) and B16 (**b**) cells following UVA, RF-EMF, and p38 inhibitor (SB203580) treatment ($n$=3). **D**. DNA damage assessment by alkaline comet assay in HaCaT and B16 cells under the same conditions with p38 inhibitor treatment ($n$=3). The $n$ value indicates the number of independent biological replicates performed on different days using separate cell passages. The data indicates the mean ± SD. Levels of statistical significance were evaluated using one-way ANOVA or unpaired Student's $t$-tests; * $p < 0.05$, ** $p < 0.01$, *** $p < 0.001$, **** $p < 0.0001$ vs. control. p38i; p38 inhibitor. Group designations are indicated by bar colors: white (control), black (RF-EMF only), red (UV only), blue (combined UV and RF-EMF), and red line (NAC or p38 inhibitor).

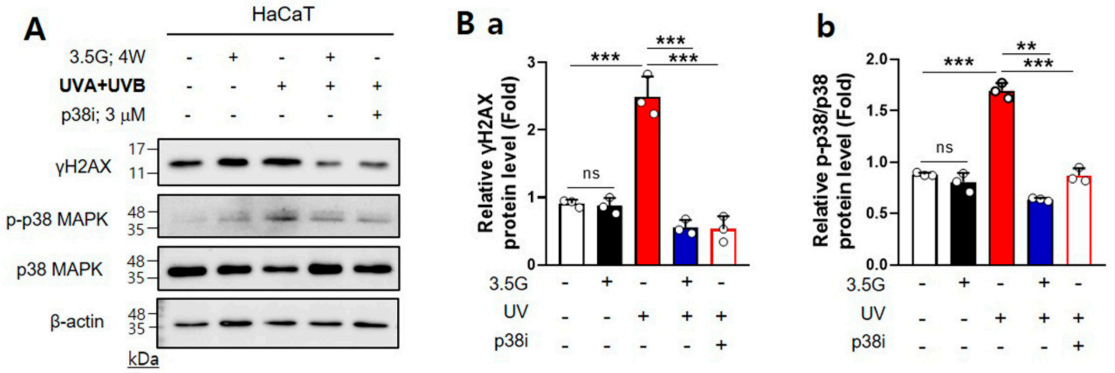


**Figure S2. Comparing the impact of a p38 inhibitor on HaCaT cell reactions to 3.5 GHz RF-EMF following UV exposure**. Cells were exposed to UV (HaCaT: 1 J/cm² UVA + 0.03 J/cm² UVB) followed by 3.5 GHz RF-EMF (4.0 W/kg, 24 h), with or without treatment of a selective p38 inhibitor (3 $\mu$M SB203580). **A.** Whole-cell lysates were subjected to SDS-PAGE and immunoblotting using antibodies against γH2AX, phospho-p38, and total p38 ($n$=3). **B.** Band intensity of γH2AX (a) and phospho-p38 (b) quantification was performed relative to total β-actin or p38 protein levels. The value $n$=3 indicates three independent biological replicates performed on different days. The data indicate the mean ± SD. Levels of statistical significance were evaluated using unpaired Student's t-tests; **p < 0.01 ***p < 0.001 vs. control. p38i; p38 inhibitor. Group designations are indicated by bar colors: white (control), black (RF-EMF only), red (UV only), blue (combined UV and RF-EMF), and red line (p38 inhibitor).
